# Relationship Between Lipoprotein (a) [Lp(a)] and Cognition in Different Ischemic Stroke Subtypes

**DOI:** 10.3389/fneur.2021.736365

**Published:** 2021-12-03

**Authors:** Jingjing Li, Shiyu Li, Yuesong Pan, Mengxing Wang, Xia Meng, Yilong Wang, Xingquan Zhao, Yongjun Wang

**Affiliations:** ^1^Department of Neurology, Beijing Tiantan Hospital, Capital Medical University, Beijing, China; ^2^China National Clinical Research Center for Neurological Diseases, Beijing Tiantan Hospital, Capital Medical University, Beijing, China

**Keywords:** lipoprotein(a), cognitive function, ischemic stroke, stroke subtypes, dementia

## Abstract

**Background and Purpose:** Although elevated serum lipoprotein (a) [Lp(a)] is considered to be a risk factor of ischemic stroke, the relationship between Lp(a) and cognitive impairment after stroke remains unclear. This study investigated the association between serum Lp(a) and cognitive function after acute ischemic stroke (AIS) or transient ischemic attack (TIA).

**Methods:** The study included 1,017 patients diagnosed with AIS or TIA from the cognition subgroup of the Third China National Stroke Registry (CNSR3). Montreal Cognitive Assessment (MoCA) at 2 weeks or discharge, 3 months, and 1 year was evaluated. The primary outcome was cognitive impairment at 1 year, defined as MoCA ≤ 22. The secondary outcome was cognition improvement at 1 year compared with 2 weeks. The association between Lp(a) levels and cognitive function was analyzed.

**Results:** Among the 1,017 patients included, 326 (32.1%) had cognitive impairment at 1 year. Patients with MoCA ≤ 22 at 1 year were older, received less education, and had higher baseline NIHSS, higher proportion of ischemic stroke history, large artery atherosclerosis (LAA) subtype, and multiple infarctions (*P* < 0.05 for all). Patients with highest Lp(a) quartile had slightly higher percentage of cognitive impairment at 1 year but without statistical difference. In subgroup analysis of LAA subtype, the patients with highest Lp(a) quartile had higher percentage of cognitive impairment at 1 year (adjusted OR:2.63; 95% CI: 1.05–6.61, *P* < 0.05). What is more, the patients with highest Lp(a) quartile in LAA subtype had lower percentage of cognition improvement at 1 year. However, similar results were not found in small artery occlusion (SAO) subtype.

**Conclusion:** Higher Lp(a) level was associated with cognitive impairment and less improvement of cognition in patients after AIS or TIA with large-artery atherosclerosis subtype.

## Introduction

Stroke and dementia are currently two main causes affecting brain function in older people (1). Stroke survivors have an increased risk of developing cognitive impairment or dementia (2–4). Studies reported that the incidence of dementia after stroke varied from 7.4% in a population-based study on first stroke to 41.3% in hospital-based cases of recurrent stroke (5). Lipoprotein (a) [Lp(a)] (6) is a low-density lipoprotein (LDL)-like particle with its apolipoprotein B100 (apoB-100) moiety linked to a large polymorphic glycoprotein named apo(a) via a single disulfide bond. Lp(a) is considered to be a risk factor of ischemic stroke because of its role in atherosclerosis and thrombosis (7). However, the relationship between serum Lp(a) and cognitive impairment or dementia is still controversial. Previous studies have shown that Lp(a) had an adverse effect (8, 9) or no effect (10–12) on cognition, whereas, in recent years some studies have found that it had a beneficial influence (13–15).

In this study, we aimed to investigate the association between serum Lp(a) levels and cognitive impairment after acute ischemic stroke (AIS) or transient ischemic attack (TIA) and analyzed it under different etiological classifications.

## Methods

### ICONS Group and Study Population

The Third China National Stroke Registry (CNSR-III) (16) is a nationwide clinical registry that includes patients who had AIS or TIA within 7 days after the onset of symptoms from 201 hospitals in China between August 2015 and January 2018. A total of 40 study sites with experience in cognition and sleep research participated in the (17) Impairment of CognitiON and Sleep quality for patients after AIS or TIA (ICON) subgroup. The inclusion criteria for ICONS were the same as those for the CNSR-III, but several exclusion criteria were added, such as prior diagnosis of cognitive impairment, schizophrenia or psychosis disease; illiteracy, and concomitant neurological disorders that interfere with cognitive or sleep evaluation, for example, severe aphasia defined as National Institutes of Health Stroke Scale (NIHSS) item 9 >2, visual impairment, hearing loss, dyslexia, severe unilateral neglect, and consciousness disorders. Patients with cerebral infarction on MRI or CT and without symptoms or signs were excluded.

### Basic Data Collection

The baseline information was collected prospectively using an electronic data capture system by face-to-face interviews, and included age, sex, body mass index (BMI), current smoking habit, medical history (hypertension, diabetes mellitus, dyslipidemia, ischemic stroke, TIA, coronary heart diseases, and atrial fibrillation/flutter), previous medication, Trial of Org 10172 in Acute Stroke Treatment (TOAST) criteria, NIHSS score, and so on.

### Measurement of Lp(a)

Fasting blood samples were collected in serum separation tubes and EDTA anticoagulation blood collection tubes within 24 h of admission from all the patients. All the blood samples were transported through cold chain to the center laboratory in Beijing Tiantan Hospital, where all serum specimens were stored at −80° refrigerator until testing was performed. Serum Lp(a) was measured by enzyme-linked immunosorbent assay (ELISA) kits (Mercodia AB, Sweden). Mercodia Lp(a) ELISA is based on the direct sandwich technique in which two monoclonal antibodies are directed against separate antigenic determinants in the Apo(a) molecule.

### Assessment of Cognition

Montreal cognitive assessment (MoCA) was evaluated by trained examiners at 2 weeks or discharge, 3 months, and 1 year after AIS or TIA. Cognitive impairment was defined as MoCA ≤ 22 at 1 year. We subtracted the MoCA values at 2 weeks from MoCA values at 1 year to evaluate cognition improvement of the patients.

### Statistical Analysis

The patients were divided into four groups by Lp(a) quartiles, and baseline characteristics were compared. Continuous variables were described by median with interquartile range (IQR) because of skewed distribution. Categorical variables were described by frequencies with percentages. Non-parametric Wilcoxon or Kruskal–Wallis test was performed to compare group differences for continuous variables, and chi-square test or Fisher exact test was performed for categorical variables. The association between Lp(a) and cognitive function was investigated with a logistic regression model. The variables were adjusted in multivariable analyses if established as a traditional predictor or with a *P*-value of ≤ 0.1 in univariate analysis. Unadjusted and adjusted odds ratios (ORs) and their 95% confidence intervals (CIs) were calculated. We also analyzed the association in different TOAST subtypes such as the large-artery atherosclerosis (LAA) and small-artery occlusion (SAO) subtypes.

Overall, a two-sided *P*-value of < 0.05 was considered statistically significant. All analyses were performed with SAS software version 9.4 (SAS Institute Inc., Cary, NC, United States).

### Data Availability Policy

The data that support the findings of this study are available from the corresponding author.

## Results

Among total 2625 patients in ICONS study, 797 patients without Lp(a) values and 550 patients without available MoCA at 1 year were excluded from this study. Thus, a total of 1,017 patients were analyzed in this study. Baseline characteristics of the excluded and included patients are presented in Supplementary Table 1. The patients were stratified into quartiles according to Lp(a) levels. The baseline characteristics are shown in Table 1. For all included patients, the median (IQR) age was 62 (53–69) years, and 743 (73.1%) of the patients were male. The median (IQR) serum Lp(a) was 145.01 (70.23–292.47) mg/dl. Compared with the lowest quartile, patients with higher quartiles of Lp(a) were older, had slightly higher proportion of ischemic stroke history and higher TC, LDL-C, TG levels (Table 1).

**Table 1:** Baseline characteristics according to Lp(a) levels.

	**Q1 (*n* = 255)**	**Q2 (*n* = 254)**	**Q3 (*n* = 253)**	**Q4 (*n* = 255)**	***P*-value**
Age, median (IQR), y	60 (52–68)	63 (54–70)	62 (56–69)	61 (54–69)	0.0497
Male (*n*, %)	193 (75.7)	181 (71.3)	174 (68.8)	195 (76.5)	0.1591
Education (*n*, %)					
Elementary	60 (24.8)	69 (27.7)	62 (25.9)	61 (25.7)	0.9884
Middle school	89 (36.8)	90 (36.1)	92 (38.5)	88 (37.1)	
High school or above	93 (38.4)	90 (36.1)	85 (35.6)	88 (37.1)	
Current smoker (*n*, %)	98 (38.4)	75 (29.5)	89 (35.2)	94 (36.9)	0.1678
Heavy drink (*n*, %)	43 (16.9)	39 (15.4)	39 (15.4)	43 (16.9)	0.9380
BMI, median (IQR)	24.6 (23.0–26.7)	24.7 (22.5–27.0)	24.9 (23.0–27.2)	24.7 (22.7–26.4)	0.5617
Medical history (*n*, %)					
Ischemic stroke	37 (14.5)	54 (21.3)	58 (22.9)	58 (22.8)	0.0593
TIA	7 (2.8)	8 (3.2)	11 (4.4)	11 (4.3)	0.6942
Hypertension	200 (78.4)	189 (74.4)	193 (76.3)	200 (78.4)	0.6563
Diabetes mellitus	90 (35.3)	97 (38.2)	79 (31.2)	95 (37.3)	0.3663
Dyslipidemia	22 (8.6)	28 (11.0)	31 (12.3)	32 (12.6)	0.4821
Coronary heart diseases	22 (8.6)	32 (12.6)	30 (11.9)	40 (15.7)	0.1116
TOAST subtypes (*n*, %)					
Large-artery atherosclerosis (LAA)	56 (22.0)	62 (24.4)	63 (24.9)	78 (30.6)	0.1697
Cardioembolism	20 (7.8)	15 (5.9)	14 (5.5)	13 (5.1)	
Small-vessel occlusion (SAO)	48 (18.8)	63 (24.8)	65 (25.7)	62 (24.3)	
Other determined etiology	2 (0.8)	4 (1.6)	2 (0.8)	0 (0.0)	
Undetermined etiology	129 (50.6)	110 (43.3)	109 (43.1)	102 (40.0)	
NIHSS, median (IQR)	2 (1–4)	3 (1–5)	3 (1–5)	3 (1–5)	0.2039
TC, median (IQR), mmol/L	3.56 (3.02–4.27)	3.94 (3.31–4.48)	4.11 (3.33–4.72)	3.97 (3.23–4.71)	<0.0001
TG, median (IQR), mmol/L	1.46 (1.10–2.08)	1.39 (1.06–1.96)	1.34 (1.04–1.73)	1.20 (0.93–1.65)	<0.0001
HDL, median (IQR), mmol/L	0.91 (0.77–1.07)	0.94 (0.77–1.13)	0.95 (0.80–1.11)	0.93 (0.80–1.14)	0.2546
LDL, median (IQR), mmol/L	1.86 (1.33–2.45)	2.26 (1.66–2.88)	2.50 (1.83–3.08)	2.43 (1.75–3.21)	<0.0001
CRP, median (IQR), mg/L	1.59 (0.83–3.23)	1.74 (0.68–4.12)	1.24 (0.68–3.48)	1.49 (0.61–4.08)	0.4193
Multiple infarctions (*n*, %)	188 (73.7)	190 (74.8)	199 (78.7)	202 (79.2)	0.3614
Cortical infarction (*n*, %)	64 (26.7)	59 (24.2)	56 (23.1)	51 (20.7)	0.5015
ICAS or ECAS (*n*, %)	113 (48.1)	117 (49.0)	118 (49.8)	131 (54.1)	0.5559

*IQR, interquartile range; BMI, body mass index; TIA, transient ischemic attack; NIHSS, National Institutes of Health Stroke Scale; TC, total cholesterol; TG, triglycerides; HDL, high-density lipoprotein; LDL, low-density lipoprotein; LDL, low-density lipoprotein; CRP, C reactive protein; ICAS, intracranial artery stenosis; ECAS, extracranial artery stenosis*.

### Lp(a) and Cognitive Function

Three hundred twenty-six (32.1%) of the patients had MoCA ≤ 22 at 1 year after AIS or TIA. Compared with patients with MoCA >22, the patients with MoCA ≤ 22 were older, had lower level of education, higher proportions of previous ischemic stroke, large-artery atherosclerosis (LAA) subtype, and multiple infarctions, and higher NIHSS and CRP levels (Supplementary Table 2).

Patients in higher quartiles of Lp(a) levels had a higher proportion of cognitive impairment at 1 year, but no statistical difference was found (the highest vs. the lowest quartile: OR 1.33; 95% CI: 0.84–2.09, *P* > 0.05). No association was found between Lp(a) and cognition improvement at 1 year compared to baseline MOCA values after AIS or TIA (Table 2).

**Table 2:** ORs of Lp (a) levels for cognitive impairment at 1 year.

**Outcomes**	**Events, *n* (%)**	**Unadjusted, OR (95%CI)**	***P*-value**	**Model 1, OR (95%CI)**	***P*-value**	**Model 2, OR (95%CI)**	***P*-value**
2 w MoCA ≤ 22							
Q1	112 (43.9)	Reference		Reference		Reference	
Q2	132 (52.0)	1.38 (0.98–1.96)	0.0695	1.29 (0.87–1.91)	0.2031	1.31 (0.87–1.97)	0.1946
Q3	124 (49.1)	1.23 (0.87–1.74)	0.2503	1.00 (0.67–1.49)	0.9864	1.02 (0.67–1.55)	0.9205
Q4	134 (52.6)	1.41 (0.998–2.00)	0.0515	1.18 (0.79–1.77)	0.4177	1.28 (0.84–1.94)	0.2552
3 m MoCA ≤ 22							
Q1	70 (28.9)	Reference		Reference		Reference	
Q2	86 (35.1)	1.33 (0.91–1.95)	0.1446	1.20 (0.78–1.86)	0.4129	1.17 (0.75–1.84)	0.4925
Q3	76 (31.9)	1.15 (0.78–1.70)	0.4741	1.06 (0.68–1.67)	0.7882	1.05 (0.66–1.68)	0.8376
Q4	85 (34.7)	1.31 (0.89–1.91)	0.1723	1.12 (0.71–1.76)	0.6249	1.12 (0.70–1.78)	0.6343
1 y MoCA ≤ 22							
Q1	65 (25.5)	Reference		Reference		Reference	
Q2	82 (32.3)	1.39 (0.95–2.05)	0.0915	1.26 (0.81–1.98)	0.3049	1.18 (0.76–1.84)	0.4699
Q3	88 (34.8)	1.56 (1.06–2.29)	0.0229	1.29 (0.83–2.03)	0.2605	1.41 (0.90–2.22)	0.1324
Q4	91 (35.7)	1.62 (1.11–2.37)	0.0128	1.22 (0.77–1.92)	0.3931	1.33 (0.84–2.09)	0.2254
1 y−2 w MoCA (%)							
≥10%							
Q1	123 (48.2)	Reference		Reference		Reference	
Q2	125 (49.2)	1.04 (0.74–1.47)	0.8253	0.90 (0.62–1.32)	0.6048	0.92 (0.62–1.36)	0.6619
Q3	116 (45.9)	0.91 (0.64–1.29)	0.5910	0.77 (0.52–1.13)	0.1785	0.76 (0.51–1.15)	0.1915
Q4	124 (48.6)	1.02 (0.72–1.44)	0.9294	0.87 (0.58–1.28)	0.4698	0.89 (0.59–1.34)	0.5713
≥20%							
Q1	85 (33.3)	Reference		Reference		Reference	
Q2	95 (37.4)	1.20 (0.83–1.72)	0.3373	1.05 (0.70–1.57)	0.8180	1.15 (0.76–1.76)	0.5083
Q3	75 (29.6)	0.84 (0.58–1.23)	0.3710	0.69 (0.45–1.06)	0.0918	0.69 (0.44–1.09)	0.1093
Q4	86 (33.7)	1.02 (0.71–1.47)	0.9253	0.85 (0.56–1.30)	0.4478	0.93 (0.60–1.44)	0.7340
≥30%							
Q1	51 (20.0)	Reference		Reference		Reference	
Q2	68 (26.8)	1.46 (0.97–2.21)	0.0719	1.36 (0.86–2.15)	0.1953	1.38 (0.85–2.22)	0.1934
Q3	48 (19.0)	0.94 (0.60–1.45)	0.7702	0.73 (0.44–1.21)	0.2190	0.66 (0.39–1.13)	0.1267
Q4	66 (25.9)	1.40 (0.92–2.12)	0.1150	1.10 (0.68–1.78)	0.7136	1.12 (0.68–1.84)	0.6682
≥40%							
Q1	37 (14.5)	Reference		Reference		Reference	
Q2	54 (21.3)	1.59 (1.00–2.52)	0.0480	1.64 (0.99–2.73)	0.0574	1.68 (0.99–2.83)	0.0545
Q3	34 (13.4)	0.92 (0.55–1.51)	0.7284	0.77 (0.43–1.37)	0.3722	0.73 (0.40–1.35)	0.3181
Q4	50 (19.6)	1.44 (0.90–2.29)	0.1272	1.28 (0.75–2.20)	0.3630	1.31 (0.75–2.27)	0.3481
≥50%							
Q1	26 (10.2)	Reference		Reference		Reference	
Q2	41 (16.1)	1.70 (1.00–2.87)	0.0490	1.79 (1.01–3.20)	0.0478	1.88 (1.04–3.41)	0.0382
Q3	28 (11.1)	1.10 (0.62–1.93)	0.7502	0.89 (0.46–1.71)	0.7198	0.83 (0.41–1.64)	0.5836
Q4	41 (16.1)	1.69 (0.998–2.85)	0.0510	1.36 (0.74–2.50)	0.3273	1.35 (0.72–2.53)	0.3530

*Model 1: adjusted for age, sex, education, previous history of ischemic stroke, hypertension, diabetes mellitus, dyslipidemia, TOAST, LDL, NIHSS, Statin therapy*.

*Model 2: adjusted for Model 1+ ICAS or ECAS, multiple infarctions, cortical infarction*.

### Subgroup Analysis in TOAST Subtypes

The median (IQR) serum Lp(a) levels in LAA and small-vessel occlusion (SAO) subtypes were 160.27(80.81–336.76) and 150.7 (79.67–303.78) mg/dl, respectively. In patients with LAA subtype, higher Lp(a) levels were associated with increased risk of cognitive impairment (MOCA ≤ 22) at 1 year. The adjusted OR for the highest vs. the lowest quartile of Lp(a) was 2.63 (95% CI 1.05–6.61, *P* < 0.05) after adjusting for potential confounding factors. What is more, higher Lp(a) quartiles were associated with lower percentage of cognition improvement at 1 year compared to cognition status at 2 weeks in LAA subtype. Adjusted ORs (95% CI) for the highest vs. lowest quartile of Lp(a) were 0.4 (0.16–0.99) and 0.22 (0.08–0.63) when cognition improvement was defined as ≥20 and 30%, respectively (Table 3). However, similar association was not found in patients with SAO subtype (Table 4).

**Table 3:** Subgroup analysis in patients with large-artery atherosclerosis (LAA) subtype.

**Outcomes**	**Events, *n* (%)**	**Unadjusted, OR (95%CI)**	***P*-value**	**Model 1, OR (95%CI)**	***P*-value**	**Model 2, OR (95%CI)**	***P*-value**
2 w MoCA ≤ 22							
Q1	35 (54.7)	Reference		Reference		Reference	
Q2	37 (56.9)	1.10 (0.55–2.19)	0.7981	0.87 (0.38–2.01)	0.7487	1.07 (0.45–2.57)	0.8731
Q3	36 (55.4)	1.03 (0.51–2.06)	0.9363	0.73 (0.31–1.70)	0.4638	0.80 (0.34–1.92)	0.6193
Q4	37 (56.9)	1.10 (0.55–2.19)	0.7981	0.93 (0.39–2.18)	0.8582	1.10 (0.45–2.69)	0.8364
3 m MoCA ≤ 22							
Q1	24 (40.0)	Reference		Reference		Reference	
Q2	20 (33.3)	0.75 (0.36–1.58)	0.4491	0.52 (0.21–1.27)	0.1509	0.51 (0.20–1.32)	0.1658
Q3	27 (43.6)	1.16 (0.56–2.38)	0.6913	0.65 (0.26–1.60)	0.3454	0.64 (0.25–1.64)	0.3519
Q4	27 (42.9)	1.13 (0.55–2.31)	0.7479	0.87 (0.35–2.15)	0.7650	0.90 (0.35–2.31)	0.8274
1 y MoCA ≤ 22							
Q1	20 (31.3)	Reference		Reference		Reference	
Q2	18 (27.7)	0.84 (0.40–1.80)	0.6581	0.72 (0.30–1.74)	0.4666	0.70 (0.28–1.75)	0.4439
Q3	27 (41.5)	1.56 (0.76–3.22)	0.2259	1.27 (0.53–3.00)	0.5940	1.27 (0.52–3.09)	0.6016
Q4	33 (50.8)	2.27 (1.11–4.65)	0.0254	2.65 (1.10–6.41)	0.0306	2.63 (1.05–6.61)	0.0397
1 y−2 w MoCA ≥ 10%							
Q1	35 (54.7)	Reference		Reference		Reference	
Q2	38 (58.5)	1.17 (0.58–2.34)	0.6655	0.87 (0.39–1.92)	0.7232	1.07 (0.46–2.47)	0.8758
Q3	32 (49.2)	0.80 (0.40–1.61)	0.5353	0.59 (0.26–1.32)	0.1988	0.53 (0.23–1.23)	0.1393
Q4	33 (50.8)	0.85 (0.43–1.71)	0.6559	0.63 (0.28–1.43)	0.2698	0.68 (0.29–1.60)	0.3749
1 y−2 w MoCA ≥ 20%							
Q1	28 (43.8)	Reference		Reference		Reference	
Q2	27 (41.5)	0.91 (0.46–1.84)	0.7996	0.68 (0.30–1.53)	0.3480	0.81 (0.35–1.90)	0.6288
Q3	23 (35.4)	0.70 (0.35–1.43)	0.3319	0.45 (0.19–1.08)	0.0738	0.42 (0.17–1.05)	0.0622
Q4	22 (33.9)	0.66 (0.32–1.34)	0.2493	0.37 (0.15–0.90)	0.0273	0.40 (0.16–0.99)	0.0473
1 y−2 w MoCA ≥ 30%							
Q1	22 (34.4)	Reference		Reference		Reference	
Q2	20 (30.8)	0.85 (0.41–1.77)	0.6622	0.51 (0.21–1.26)	0.1465	0.56 (0.22–1.42)	0.2201
Q3	19 (29.2)	0.79 (0.38–1.66)	0.5307	0.44 (0.17–1.13)	0.0871	0.39 (0.15–1.05)	0.0628
Q4	14 (21.5)	0.52 (0.24–1.15)	0.1067	0.22 (0.08–0.61)	0.0036	0.22 (0.08–0.63)	0.0050
1 y−2 w MoCA ≥ 40%							
Q1	15 (23.4)	Reference		Reference		Reference	
Q2	16 (24.6)	1.07 (0.48–2.39)	0.8756	0.89 (0.34–2.28)	0.8010	0.89 (0.34–2.35)	0.8200
Q3	9 (13.9)	0.53 (0.21–1.31)	0.1657	0.31 (0.10–0.99)	0.0480	0.29 (0.09–0.95)	0.0408
Q4	11 (16.9)	0.67 (0.28–1.59)	0.3582	0.51 (0.18–1.47)	0.2147	0.52 (0.18–1.52)	0.2304
1 y−2 w MoCA ≥ 50%							
Q1	11 (17.2)	Reference		Reference		Reference	
Q2	12 (18.5)	1.09 (0.44–2.69)	0.8500	0.92 (0.32–2.64)	0.8825	0.94 (0.32–2.74)	0.9037
Q3	8 (12.3)	0.68 (0.25–1.81)	0.4364	0.38 (0.11–1.38)	0.1409	0.38 (0.10–1.40)	0.1459
Q4	10 (15.4)	0.88 (0.34–2.23)	0.7816	0.61 (0.19–1.94)	0.4036	0.64 (0.20–2.10)	0.4641

*Model 1: adjusted for age, sex, education, previous history of ischemic stroke, hypertension, diabetes mellitus, dyslipidemia, TOAST, LDL, NIHSS, Statin therapy*.

*Model 2: adjusted for Model 1+ ICAS or ECAS, multiple infarctions, cortical infarction*.

**Table 4:** Subgroup analysis in patients with small-vessel occlusion (SAO) subtype.

**Outcomes**	**Events, *n* (%)**	**Unadjusted, OR (95%CI)**	***P*-value**	**Model 1, OR (95%CI)**	***P*-value**	**Model 2, OR (95%CI)**	***P*-value**
2 w MoCA ≤ 22							
Q1	18 (30.5)	Reference		Reference		Reference	
Q2	30 (50.0)	2.28 (1.08–4.82)	0.0316	1.73 (0.71–4.19)	0.2278	1.91 (0.77–4.72)	0.1638
Q3	25 (42.4)	1.68 (0.79–3.57)	0.1822	0.96 (0.38–2.45)	0.9301	1.01 (0.39–2.64)	0.9803
Q4	29 (48.3)	2.13 (1.01–4.51)	0.0483	1.27 (0.52–3.14)	0.5992	1.31 (0.52–3.27)	0.5652
3 m MoCA ≤ 22							
Q1	11 (20.8)	Reference		Reference		Reference	
Q2	17 (29.3)	1.58 (0.66–3.79)	0.3021	1.29 (0.49–3.43)	0.6108	1.25 (0.46–3.41)	0.6680
Q3	18 (31.6)	1.76 (0.74–4.19)	0.2008	1.60 (0.59–4.34)	0.3568	1.57 (0.56–4.38)	0.3915
Q4	17 (29.8)	1.62 (0.68–3.89)	0.2776	1.07 (0.39–2.90)	0.9025	1.02 (0.37–2.83)	0.9705
1 y MoCA ≤ 22							
Q1	10 (17.0)	Reference		Reference		Reference	
Q2	20 (33.3)	2.45 (1.03–5.83)	0.0427	2.11 (0.78–5.76)	0.1437	2.37 (0.84–6.67)	0.1017
Q3	19 (32.2)	2.33 (0.97–5.57)	0.0577	2.45 (0.87–6.88)	0.0887	2.77 (0.95–8.04)	0.0613
Q4	16 (26.7)	1.78 (0.73–4.33)	0.2027	1.12 (0.37–3.32)	0.8452	1.20 (0.39–3.65)	0.7490
1 y−2 w MoCA ≥ 10%							
Q1	26 (44.1)	Reference		Reference		Reference	
Q2	27 (45.0)	1.04 (0.50–2.14)	0.9185	0.69 (0.30–1.58)	0.3818	0.73 (0.32–1.69)	0.4631
Q3	20 (33.9)	0.65 (0.31–1.37)	0.2585	0.31 (0.12–0.79)	0.0133	0.31 (0.12–0.79)	0.0143
Q4	27 (45.0)	1.04 (0.50–2.14)	0.9185	0.59 (0.25–1.39)	0.2251	0.61 (0.26–1.46)	0.2667
1 y−2 w MoCA ≥ 20%							
Q1	16 (27.1)	Reference		Reference		Reference	
Q2	18 (30.0)	1.15 (0.52–2.55)	0.7280	0.63 (0.24–1.64)	0.3441	0.70 (0.26–1.87)	0.4791
Q3	14 (23.7)	0.84 (0.37–1.92)	0.6727	0.36 (0.12–1.07)	0.0657	0.40 (0.13–1.21)	0.1027
Q4	20 (33.3)	1.34 (0.61–2.95)	0.4611	0.79 (0.30–2.05)	0.6243	0.86 (0.32–2.29)	0.7620
1 y−2 w MoCA ≥ 30%							
Q1	7 (11.9)	Reference		Reference		Reference	
Q2	10 (16.7)	1.49 (0.53–4.21)	0.4560	1.12 (0.33–3.82)	0.8509	1.09 (0.31–3.82)	0.8914
Q3	9 (15.3)	1.34 (0.46–3.86)	0.5916	0.45 (0.10–1.96)	0.2870	0.43 (0.10–1.93)	0.2708
Q4	16 (26.7)	2.70 (1.02–7.16)	0.0457	2.15 (0.65–7.04)	0.2081	2.05 (0.61–6.89)	0.2439
1 y−2 w MoCA ≥ 40%							
Q1	6 (10.2)	Reference		Reference		Reference	
Q2	9 (15.0)	1.56 (0.52–4.69)	0.4299	1.27 (0.35–4.61)	0.7223	1.28 (0.34–4.80)	0.7156
Q3	6 (10.2)	1.00 (0.30–3.30)	1.0000	0.45 (0.09–2.24)	0.3305	0.45 (0.09–2.27)	0.3307
Q4	11 (18.3)	1.98 (0.68–5.77)	0.2089	1.61 (0.43–6.06)	0.4826	1.60 (0.42–6.18)	0.4933
1 y−2 w MoCA ≥ 50%							
Q1	5 (8.5)	Reference		Reference		Reference	
Q2	7 (11.7)	1.43 (0.43–4.78)	0.5646	1.53 (0.39–6.08)	0.5434	1.59 (0.39–6.50)	0.5185
Q3	4 (6.8)	0.79 (0.20–3.08)	0.7292	0.20 (0.02–2.01)	0.1728	0.20 (0.02–2.07)	0.1783
Q4	9 (15.0)	1.91 (0.60–6.07)	0.2751	1.86 (0.44–7.88)	0.4009	1.86 (0.43–8.09)	0.4096

*Model 1: adjusted for age, sex, education, previous history of ischemic stroke, hypertension, diabetes mellitus, dyslipidemia, TOAST, LDL, NIHSS, Statin therapy*.

*Model 2: adjusted for Model 1+ ICAS or ECAS, multiple infarctions, cortical infarction*.

## Discussion

This study found that higher Lp(a) level was associated with cognitive impairment and less cognition improvement at 1 year after AIS or TIA in LAA subtype. Similar association was not found in SAO subtype.

Lipoprotein (a) [Lp(a)] was considered to be a risk factor of ischemic stroke, although the results were inconsistent. The meta-analysis indicated that elevated Lp(a) level was associated with increased risk of ischemic stroke (7). A Mendelian randomization study (18) showed that 1-SD genetically lowered Lp(a) level was associated with a 13% lower risk of stroke. Recent studies revealed that Lp(a) might play different roles in different stroke subtypes. A prospective stroke registry study in Korean (19) showed that Lp(a) levels in large artery atherosclerosis (LAA) stroke were significantly higher than other four subtypes, and that elevated Lp(a) levels were associated with extensive burden of intracranial and extracranial vascular steno-occlusive lesions. What is more, a Mendelian randomization analysis (14) based on data from a large-scale GWAS study demonstrated that higher Lp(a) levels may lead to an increased risk of large artery stroke but a decreased risk of small vessel stroke. Studies on the association between Lp(a) concentrations and cognitive impairment are limited and controversial. Many studies showed Lp(a) was higher in vascular dementia than healthy controls (20, 21). Cross-sectional data of 1,380 healthy Berlin Aging Study II (BASE-II) (8) participants suggested that men with lower Lp(a) concentrations had better cognitive performance. Conversely, a prospective cohort of 2,532 subjects of Finnish (15) male population in 24.9 years' follow-up, showed that Lp(a) was protective of future dementia risk. Another large prospective cohort study based on four United States communities (13) showed that higher Lp(a) levels were associated with slower cognitive decline in semantic fluency over 15 years. What is more, negative results were also obtained in several studies. A previous cross-sectional study (12) has found that no statistically significant difference in cognitive performances between subjects with elevated and normal Lp(a) levels and subjects who showed a similar decline rate during follow-up. A large prospective cohort study on pravastatin in the elderly at risk (PROSPER) (11) over an average 3.2 years of follow-up proved that Lp(a) was a predictor of combined cardiovascular events instead of cognitive function. However, most of these inconsistent results came from the follow-up of non-affected populations. This study focused on cognitive impairment and cognition changes after AIS or TIA events and found that higher Lp(a) levels were associated with cognitive impairment and less cognition improvement at 1 year in LAA subtype instead of SAO subtype. These results suggested that Lp(a) may play different roles in different stroke types.

Serum Lp(a) is genetically determined and relatively stable throughout the life of an individual. Lifestyle changes and conventional lipid-lowering therapies seem to be ineffective in reducing elevated Lp(a) levels. Oxidative stress and inflammation have been proved to play important roles in the pathogenesis of atherosclerosis and thrombosis (22, 23). Studies also suggested that Lp(a) was involved in atherosclerosis and thrombosis (6), although the pathophysiology remained unclear. The presence of oxidized phospholipids (OxPLs) on Lp(a) and apo(a) positive staining in human atherosclerotic plaques confers its proatherogenic property (24). Cerrato (25) found that Lp(a) was associated with large vessel disease independent of LDL level, suggesting its primary involvement in atherothrombotic stroke. The apo(a) in Lp(a) has structural similarities with plasminogen and inhibits fibrinolysis by interfering with the conversion of plasminogen to plasmin, which is attributed to the thrombotic actions of Lp(a). Studies showed that age-related loss of cognitive function might be driven by atherosclerotic effects associated with altered lipid patterns, and that optimal control of cardiovascular risk factors may reduce the risk of cognitive decline (26). Given the established role of Lp(a) levels in atherosclerosis and thrombosis, elevated Lp(a) levels may aggravate cognitive impairment by promoting the occurrence of ischemic events, which is consistent with the results in LAA subtype in this study. Research studies on Lp(a) and SAO subtype were very limited. A previous study has shown that Lp(a) promoted atherothrombotic stroke instead of lacunar stroke (25). The aforementioned Mendelian randomization study (14) proved that genetically predicted higher Lp(a) concentrations may lead to a decreased risk of small vessel stroke. This study did not find similar adverse effects of Lp(a) on cognition in SAO subtype, which suggested that there might exist different mechanisms compared with LAA subtype. Further exploration is needed on the relationship between Lp(a) and different stroke subtypes, especially in SAO subtype.

This study has several strengths. First, we analyzed the relationship between Lp(a) and cognition after AIS or TIA in different stroke subtypes, which is very limited so far. Second, this study was based on a large stroke registration study, and patients were evaluated for cognitive function at baseline (after stroke events), 3 months, and 1 year after AIS or TIA, which allowed us to dynamically analyze the changes in cognition. However, this study also has limitations: First, the patients selected in this study had mainly mild ischemic stroke or patients with TIA, which might not represent all patients with AIS/TIA. Second, more than half of the patients were excluded because of missing Lp(a) or MoCA variables, although the baseline characteristics of the excluded and included patients were well-balanced. Third, MoCA required a relatively higher degree of cooperation, although the ICONS excluded prior diagnosis of cognitive impairment, psychosis disease, illiterate patients, severe aphasia, and so on.

## Conclusion

Higher Lp(a) level was associated with cognitive impairment and less improvement of cognition in patients after AIS or TIA with large-artery atherosclerosis subtype.

## Data Availability Statement

The raw data supporting the conclusions of this article will be made available by the authors, without undue reservation.

## Ethics Statement

The studies involving human participants were reviewed and approved by IRB approval number: KY2015-001-01. The patients/participants provided their written informed consent to participate in this study.

## Author Contributions

JL and SL conducted this study, interpreted the data, and drafted the manuscript. YoW designed and supervised this study and interpreted the data. XZ, YiW, and XM supervised this study. MW and YP conducted the statistical analysis and interpreted the data. SL conducted this study and statistical analysis and interpreted the data. All authors contributed to the article and approved the submitted version.

## Funding

This study was supported by the Ministry of Science and Technology of the People's Republic of China (Grant Nos: 2016YFC0901001, 2016YFC0901002, 2017YFC1310901, and 2017YFC1310902) and Beijing Municipal Science & Technology Commission (Grant No: D151100002015003).

## Conflict of Interest

The authors declare that the research was conducted in the absence of any commercial or financial relationships that could be construed as a potential conflict of interest.

## Publisher's Note

All claims expressed in this article are solely those of the authors and do not necessarily represent those of their affiliated organizations, or those of the publisher, the editors and the reviewers. Any product that may be evaluated in this article, or claim that may be made by its manufacturer, is not guaranteed or endorsed by the publisher.
